# Mutational and Combinatorial Control of Self-Assembling and Disassembling of Human Proteasome α Subunits

**DOI:** 10.3390/ijms20092308

**Published:** 2019-05-09

**Authors:** Taichiro Sekiguchi, Tadashi Satoh, Eiji Kurimoto, Chihong Song, Toshiya Kozai, Hiroki Watanabe, Kentaro Ishii, Hirokazu Yagi, Saeko Yanaka, Susumu Uchiyama, Takayuki Uchihashi, Kazuyoshi Murata, Koichi Kato

**Affiliations:** 1 School of Physical Science, SOKENDAI (The Graduate University for Advanced Studies), Okazaki, Aichi 444-8787, Japan; sekiguchi@ims.ac.jp (T.S.); saeko-yanaka@ims.ac.jp (S.Y.); 2 Institute for Molecular Science, National Institutes of Natural Sciences, 5-1 Higashiyama, Myodaiji, Okazaki, Aichi 444-8787, Japan; 3 Exploratory Research Center on Life and Living Systems (ExCELLS), National Institutes of Natural Sciences, 5-1 Higashiyama, Myodaiji, Okazaki, Aichi 444-8787, Japan; hwatanabe@d.phys.nagoya-u.ac.jp (H.W.); ishii@ims.ac.jp (K.I.); suchi@bio.eng.osaka-u.ac.jp (S.U.); uchihast@d.phys.nagoya-u.ac.jp (T.U.); 4 Graduate School of Pharmaceutical Sciences, Nagoya City University, 3-1 Tanabe-dori, Mizuho-ku, Nagoya 467-8603, Japan; tadashisatoh@phar.nagoya-cu.ac.jp (T.S.); hyagi@phar.nagoya-cu.ac.jp (H.Y.); 5 Faculty of Pharmacy, Meijo University, Tempaku-ku, Nagoya 468-8503, Japan; kurimoto@meijo-u.ac.jp; 6 National Institute for Physiological Sciences, National Institutes of Natural Sciences, 5-1 Higashiyama, Myodaiji, Okazaki, Aichi, 444-8787, Japan; chsong@nips.ac.jp (C.S.); kazum@nips.ac.jp (K.M.); 7 Department of Physics, Nagoya University, Furo-cho, Chikusa-ku, Nagoya, 464-8602, Japan; toshiya.kozai@unibas.ch; 8 Department of Biotechnology, Graduate School of Engineering, Osaka University, 2-1 Yamadaoka, Suita, Osaka 565-0871, Japan; 9 School of Life Science, SOKENDAI (The Graduate University for Advanced Studies), Okazaki, Aichi 444-8787, Japan

**Keywords:** proteasome, self-assembly, homo-oligomer, hetero-oligomer, size exclusion chromatography, native mass spectrometry, crystal structure, atomic force microscopy, electron microscopy

## Abstract

Eukaryotic proteasomes harbor heteroheptameric α-rings, each composed of seven different but homologous subunits α1–α7, which are correctly assembled via interactions with assembly chaperones. The human proteasome α7 subunit is reportedly spontaneously assembled into a homotetradecameric double ring, which can be disassembled into single rings via interaction with monomeric α6. We comprehensively characterized the oligomeric state of human proteasome α subunits and demonstrated that only the α7 subunit exhibits this unique, self-assembling property and that not only α6 but also α4 can disrupt the α7 double ring. We also demonstrated that mutationally monomerized α7 subunits can interact with the intrinsically monomeric α4 and α6 subunits, thereby forming heterotetradecameric complexes with a double-ring structure. The results of this study provide additional insights into the mechanisms underlying the assembly and disassembly of proteasomal subunits, thereby offering clues for the design and creation of circularly assembled hetero-oligomers based on homo-oligomeric structural frameworks.

## 1. Introduction

Proteins in living systems are often assembled into filamentous and circular oligomers, which exert appropriate biological functions and are deposited as malfunctional aggregates, such as pathological amyloids. Circular assemblages composed of identical protomers give rise to functional barrels or cages, as exemplified by chaperonins and AAA ATPases [1,2,3]. The design and creation of circular oligomers have been important and challenging issues in protein engineering [4,5]. During evolutionary processes, the building blocks of these homo-oligomers acquire diversity in sequence and structure, maintaining their assembling properties. The proteasome system is one of the best examples demonstrating this concept [6,7,8].

The proteasome is a huge protein complex harboring a proteolytic chamber termed the 20S core particle. This 20S core particle comprises the α- and β-rings, two types of heptameric rings arranged as a cylindrical, four-layered αββα structure [6,7,8,9,10,11,12,13]. In the archaea, the heptameric α-ring is composed of seven identical α subunits and the heptameric β-ring is composed of one or two kinds of β subunits. A total of 28 subunits are spontaneously assembled into the 20S core particle [14]. In contrast, in eukaryotes, both the α- and the β-rings are heteroheptamers composed of seven different but homologous subunits, i.e., α1–α7 and β1–β7. Assembly of these subunits is not an autonomous process but is assisted by several chaperones operating as molecular matchmakers and checkpoints [8,11,13,15,16,17,18,19,20,21].

Among all the human proteasome α subunits, α7 exhibits a unique feature of in vitro self-assembly into a homotetradecameric double-ring structure [22,23,24,25], thereby raising the question whether the α7 homotetradecamer is an off-pathway dead-end product of the proteasome formation process. Another possibility is that certain mechanisms exist for disassembling the homo-oligomer of the α7 subunit, thereby resulting in its monomeric form, which is a component of the heteroheptameric α-ring.

We previously determined a crystal structure of the human α7 homotetradecamer and demonstrated that α6, which exists as a monomer, interacts with this assemblage, thereby disrupting its double-ring structure [25,26]. These findings suggest that different α subunits have different assembly properties. We comprehensively characterized the oligomeric states of the α subunits of the human proteasome and examined their possible interplay for obtaining a better understanding of the design principles underlying the self-assembly and disassembly of the proteasomal subunits, and more generally, those behind formation of circularly assembled hetero-oligomers composed of structurally homologous subunits.

## 2. Results and Discussion

### 2.1. Oligomeric States of Human Proteasomal α Subunits

**Figure 1:**
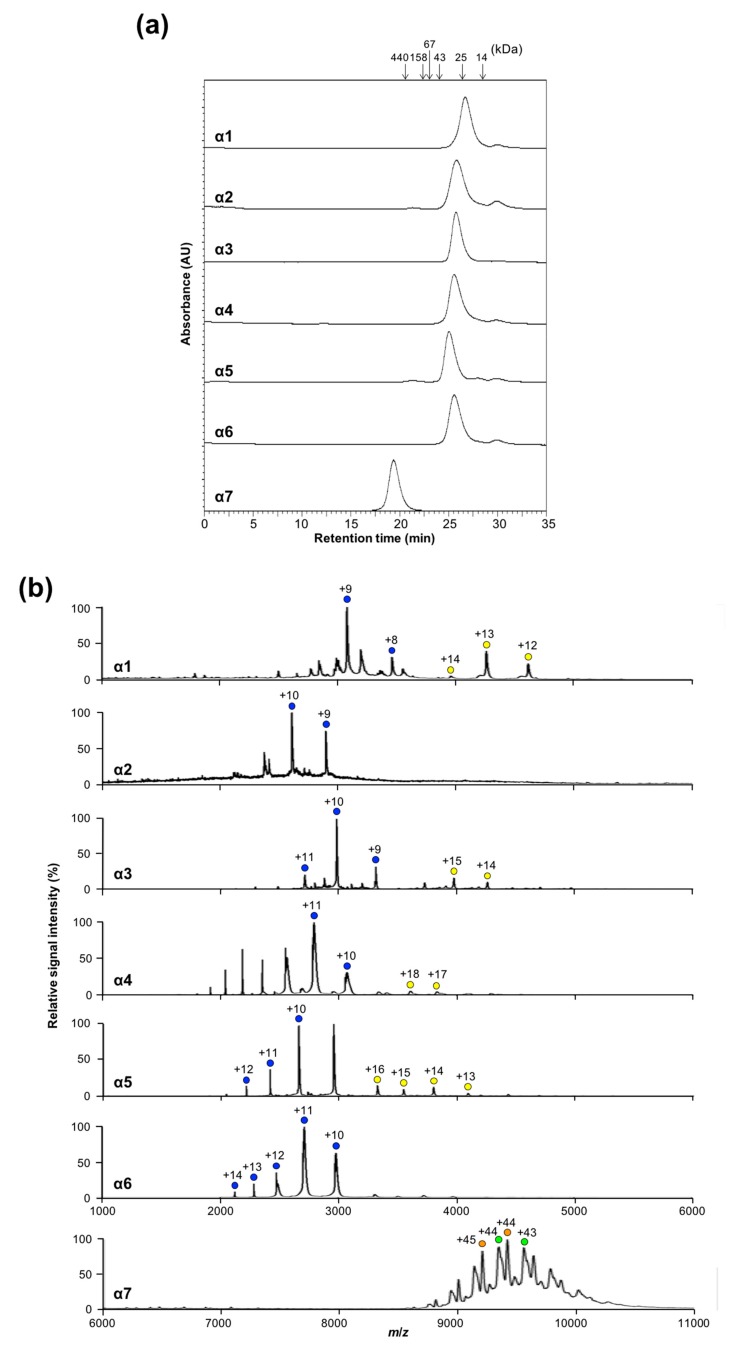
Characterization of the oligomeric states of proteasome α subunits. (**a**) Size-exclusion chromatogram of α1–α7 subunits. Arrows indicate eluted positions of the size markers. (**b**) Mass spectra of α1–α7 subunits under nondenaturing conditions. Blue and yellow circles indicate the ion series of α1–α6 monomers and dimers, respectively. Green and orange circles indicate the ion series of the α7 homotetradecamer. The estimated molecular masses of the α1–α7 subunits are as follows: 27,688.97 ± 9.91 and 55,398.59 ± 3.26 (α1 dimer and monomer, respectively); 26,034.50 ± 4.01 (α2); 59,650.89 ± 6.81 and 29,818.58 ± 0.72 (α3); 61,304.84 ± 9.52 and 30,669.77 ± 0.02 (α4); 53,162.75 ± 1.93 and 26,559.17 ± 1.39 (α5); 29,696.61 ± 9.90 (α6); and 414,406.97 ± 51.59 and 411,316.63 ± 48.20 (α7).

To characterize the oligomeric states of the α subunits in solution, we performed size-exclusion chromatography (SEC) and native mass spectrometry (MS). SEC data revealed that the major fractions of α1, α2, α3, α4, α5, and α6 corresponded to monomeric-to-dimeric forms and that α7 formed a significantly larger oligomer, as shown in Figure 1a. These findings were confirmed by native MS, which indicated that α7 existed as a homotetradecamer and that the remaining subunits exhibited one major and one minor ion series, corresponding to the molecular masses of the monomer and dimer, respectively, as shown in Figure 1b. Based on these data and our previously reported data regarding sedimentation velocity analytical ultracentrifugation [25], we concluded that among the seven types of α subunits, only α7 is self-assembled into the homotetradecameric double-ring structure and that the remaining α subunits are under equilibrium between the monomeric and dimeric forms.

### 2.2. Mutational Disassembling of the α7 Homotetradecamer

**Figure 2:**
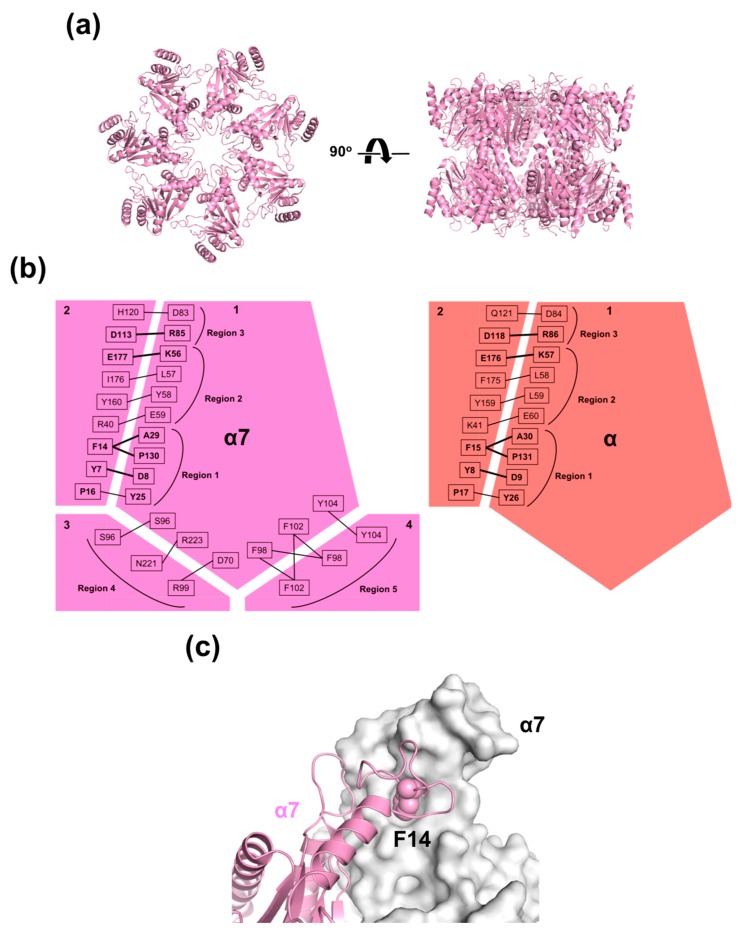
Schematic representation of the binding interfaces of the α7 homotetradecamer and the archaeal α homoheptamer. (**a**) Crystal structure of the α7 homotetradecamer (Protein Data Bank (PDB) code: 5DSV). The left and right structures are related by a rotation of 90° around the horizontal axis. (**b**) Schematic representations of intersubunit *cis* and *trans* interactions of the human α7 homotetradecamer (adjacent four molecules) together with *cis* interactions of the *Archaeoglobus fulgidus* proteasome α homoheptamer (adjacent two molecules, PDB code: 1J2P). Interaction pairs conserved between human α7 and *A. fulgidus* proteasome α subunits are highlighted by bold lines. (**c**) Surface and ribbon models of intersubunit *cis* interaction highlighting F14 residue in α7 homotetradecamer.

**Figure 3:**
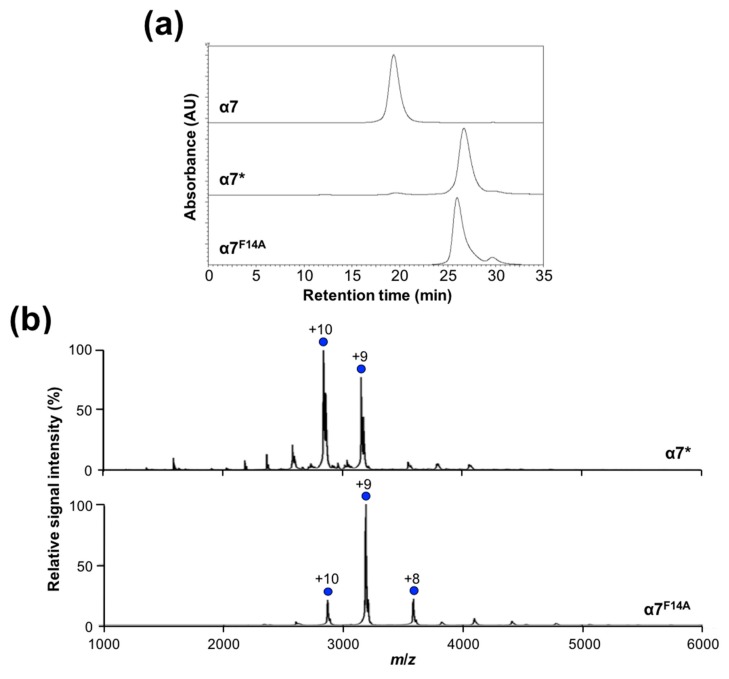
Characterization of the oligomeric states of α7 monomeric mutants. (**a**) Size-exclusion chromatogram of α7* and α7^F14A^ together with wild-type α7. (**b**) Mass spectra of α7* and α7^F14A^ under nondenaturing conditions. Blue circles indicate the ion series of the α7* or α7^F14A^ monomer. The mass spectra of α7* and α7^F14A^ mutants under nondenaturing conditions exhibited the major ion series with molecular masses of the monomer 28,456 ± 0 (with a theoretical mass: 28,594 Da) and 28,691 ± 0.05 (28,638 Da), respectively.

To gain insight into the mechanisms of the assembly of α7, we inspected the crystal structure of its homotetradecamer [25]; our inspection highlighted five regions (regions 1–5) mediating intersubunit interactions, as shown in Figure 2. Regions 1, 2, and 3 are involved in intra-ring (*cis*) interactions, which are shared with the homoheptameric α-ring of archaeal 20S core particle [9,27], whereas regions 4 and 5 are involved in inter-ring (*trans*) interactions. Comparison of amino acid sequences across the human α subunits in terms of these regions revealed that region 1 involves highly conserved contacting pairs of residues (P16–Y25, F14–A29, and F14–P130), whereas most other pairs are unique for α7, which explains its specific self-assembling property, as shown in Figure S1 and Table S1. Intriguingly, mutational monomerization of an archaeal α subunit (*Thermoplasma acidophilum*) was achieved by truncating region 1 (residues 2–34), which was accompanied by alanine substitutions of arginine residues (Arg57, Arg86, and Arg130) in regions 2 and 3 [14]. Therefore, we tested whether the conserved residues in region 1 contribute to the formation of the human α7 homo-oligomer using an α7 mutant (α7*) in which the N-terminal segment in region 1 (residues 1–22, MSYDRAITVFSPDGHLFQVEYA) was replaced with a hexahistidine-containing segment (MGSSHHHHHHSSGLVPRGSHMGS). SEC and native MS indicated that α7* was monomeric in solution, as shown in Figure 3, thereby demonstrating that deletion of the N-terminal segment disrupts the homotetradecameric structure. Close inspection of region 1 in the crystal structure of the α7 homotetradecamer highlighted the hydrophobic ball-and-socket joint composed of Phe14 in one subunit fitting into a socket formed by Ala29 and Pro130 in the neighboring subunit, as shown in Figure 2c. A single-mutation F14A disassembled the α7 tetradecamer into the monomeric form, as shown in Figure 3, indicating that the N-terminal segment, particularly Phe14, is critically involved in intra-ring *cis* interaction as a prerequisite for inter-ring *trans* interaction. Because Phe14 as well as Ala29 and Pro130 in region 1 are all conserved across α1–α7, the remaining regions, i.e., regions 2–5, are likely to reinforce the α7-specific homophilic interaction, thereby stabilizing the tetradecameric complex.

The α7 tetradecamer involves inter-ring interactions mediated through regions 4 and 5. In contrast, despite the fact that like α7, *T. acidophilum* formed a double-ring structure in solution, the α subunit of the *Archaeoglobus fulgidus* proteasome exhibits a single-ring structure in the crystal [14,27]. We previously reported that the α7 single-ring structure can be stabilized on a mica surface and that the α7 tetradecameric double-ring structure is disassembled upon addition of the α6 subunit, thereby forming a 1:7 hetero-octameric α6/α7 complex [25,26]. These observations suggest that the inter-ring *trans* interaction is dispensable for stabilizing the heptameric ring structure. We examined this possibility via a mutational approach.

**Figure 4:**
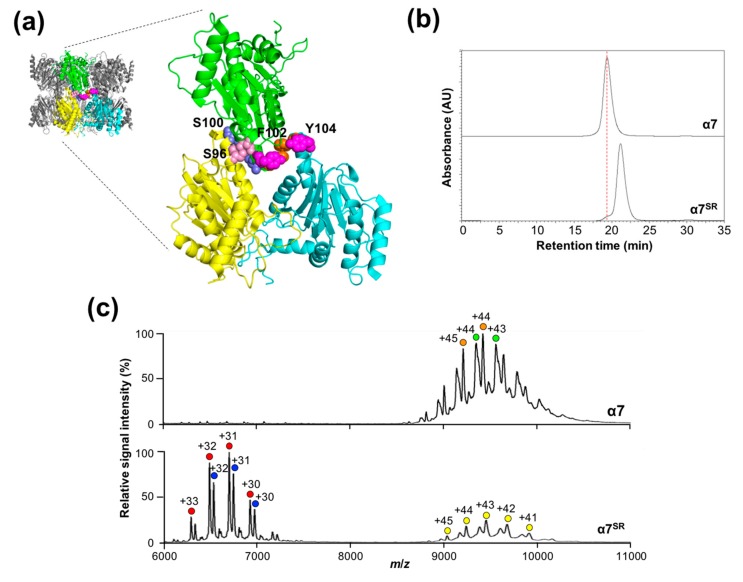
Generation of the single-ring α7 mutant. (**a**) Mutated positions of the single-ring mutant (α7^SR^). The mutated resides shown in sphere models are labeled in the close-up view. (**b**) Size-exclusion chromatogram of α7^SR^. Red dotted line indicates position of the SEC peak of α7 homotetradecamer. (**c**) Mass spectra of α7^SR^ under nondenaturing conditions. Green and orange circles indicate the ion series of the α7 homotetradecamer. Blue and red circles indicate the ion series of the homoheptameric complex of α7^SR^. Yellow circles show those of the homotetradecameric complex of α7^SR^.

For this purpose, we attempted to introduce electrostatic repulsion at the double-ring interface by focusing on the three autologously contacting pairs Ser96–Ser96, Phe102–Phe102, and Tyr104–Tyr104. Ser96 was substituted with aspartate, whereas Phe102 and Tyr104 were both substituted with arginine, as shown in Figure 4a. In addition to these positions, because the Ser100 positions are spatially proximal to each other across the inter-ring interface, Ser100 was mutated into aspartate. In SEC, the great majority of this quadruple α7 mutant (α7^SR^) eluted significantly later (at 21.1 min) than the wild-type α7 double ring (at 19.4 min), as shown in Figure 4b. Under nondenaturing conditions, the mass spectrum of this mutant exhibited a major ion series with molecular masses of 207,581 ± 78 and 209,044 ± 50 Da, corresponding to the heptameric α7 subunits (with a theoretical mass of 199,331 Da), as shown in Figure 4c. In addition, using atomic force microscopy (AFM), we confirmed the heptameric ring of the α7 mutant with a height of ~4 nm, which was half of that of the wild-type α7 double-ring (~9 nm) [26], as shown in Figure S2. All these data indicate that the double-ring tetradecamer of α7 is disassembled into a single heptameric ring by mutations bringing about electrostatic repulsion at the inter-ring interface, thereby demonstrating that the inter-ring interaction is dispensable for the formation of the homoheptameric ring of α7.

### 2.3. Disassembly of the α7 Double-Ring via Subunit Interactions

**Figure 5:**
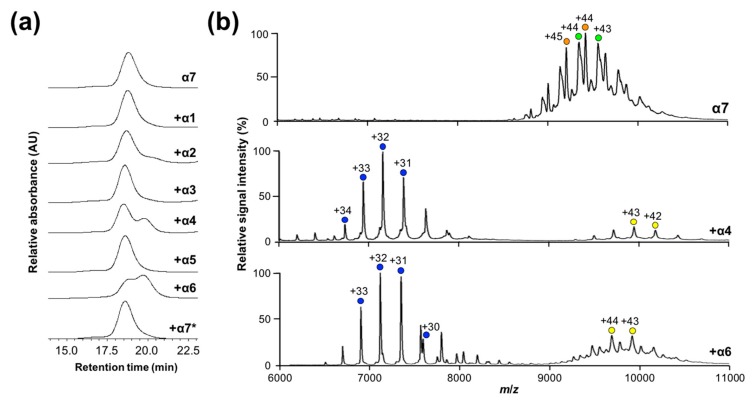
Evaluation of the α7-disassembling ability of the proteasome α subunits. (**a**) Size-exclusion chromatogram patterns of α7-containing fractions depending on the presence of the α1–α6 subunits and the α7* mutant (20 μM monomers). (**b**) Mass spectra of α4/α7 and α6/α7 mixtures under nondenaturing conditions. Green and orange circles indicate the ion series of the α7 homotetradecamer. Blue circles indicate the ion series of the 1:7 hetero-octameric complexes of α4/α7 and α6/α7, and yellow circles indicate those of the 1:14 heteropentadecameric complexes of α4/α7 and α6/α7.

In addition to being disassembled by mutations, the α7 double-ring structure can be disassembled into its single-ring structure via interaction with the monomeric α6 subunit, thereby forming a 1:7 hetero-octameric α6/α7 complex [25,26]. We examined whether the other α subunits have such a disassembling capability by performing a comprehensive analysis of the oligomeric states of α7 in the presence of the α1–α6 subunits along with the monomerized α7 mutant α7*. The SEC data indicated that although neither α1, α2, α3, α5, nor α7* affected the tetradecameric structure of α7, approximately 20% and 50% of the α7 tetradecamer was disrupted into smaller complexes by α4 or α6, respectively, judging from the peak intensity reduction, as shown in Figure 5a. The almost identical elution times (19.7 min) between the resultant complexes generated in the α4/α7 and α6/α7 mixtures implied that like α6/α7, α4 and α7 also form a 1:7 hetero-octameric complex [25,26]. To confirm this, we performed native MS of the α4/α7 mixture; results indicated that like the α6/α7 complex [25], the α4/α7 mixture exhibits a major ion series corresponding to a 1:7 hetero-octameric α4/α7 complex with a molecular mass of 228,789 ± 9 Da (theoretical molecular mass: 228,984 Da) under nondenaturing conditions, as shown in Figure 5b. The native MS data also indicated that the higher-molecular–mass complexes observed for these mixtures under the present conditions corresponded to complexes composed of fourteen α7 subunits and one α4 or α6 subunit, indicating that α4 and α6 can bind to the α7 double ring.

**Figure 6:**
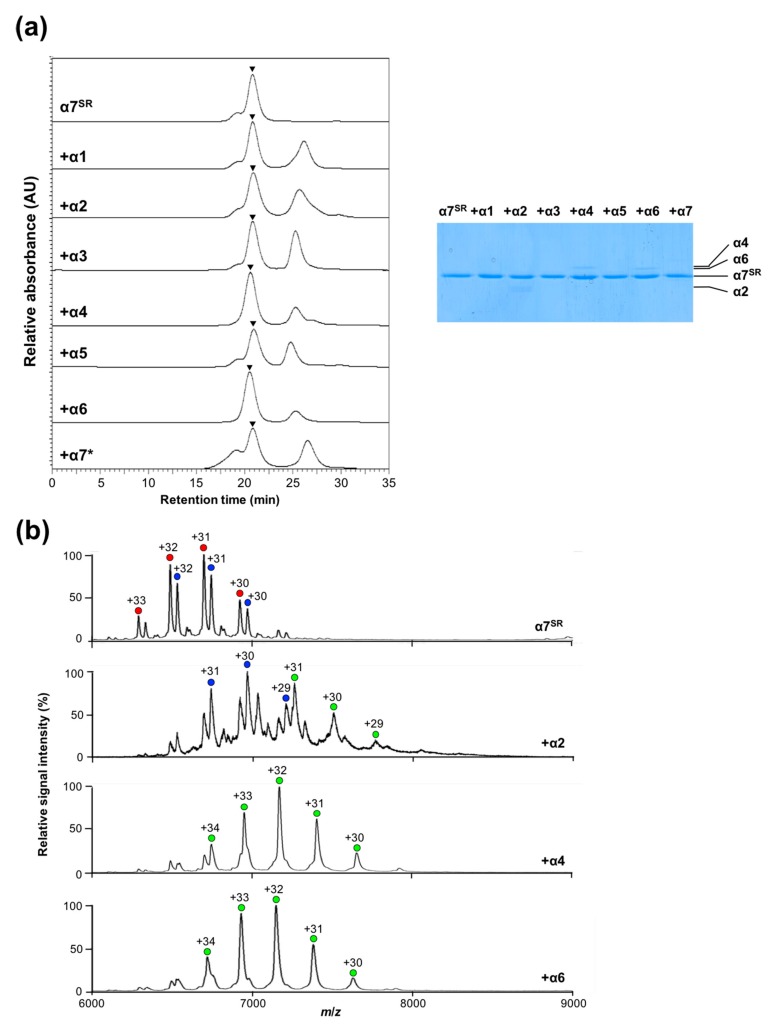
Examination of possible interactions of the α7 single ring and the proteasome α subunits. (**a**) Size-exclusion chromatogram of α7^SR^ in the presence of the α1–α6 subunits and the α7* mutant (20 μM monomers): SDS-PAGE of the size-exclusion chromatography (SEC) peaks (at approximately 21 min) originating from the α7^SR^/α1–α6 and α7* complexes. The peak position is indicated by an inverted triangle. (**b**) Mass spectra of the α7^SR^/α2, α7^SR^/α4, and α7^SR^/α6 mixtures under nondenaturing conditions. Blue and red circles indicate major ion series of the α7^SR^. Green circles show the ion series of the 7:1 hetero-octameric complexes of α7^SR^/α2, α7^SR^/α4, and α7^SR^/α6 complexes.

Our previous AFM data indicated that the disassembly of the α7 double ring by α6 involves two steps—the α6 monomer initially cracks at the interface between two stacked α7 single rings and subsequently occupies the central pore of the α7 single ring [26]. Therefore, we examined possible interactions of the α7 single ring with α4 as well as α6. Using the α7^SR^ mutant, we performed SEC-based binding analysis with the α1–α6 subunits together with α7*, as shown in Figure 6a. Sodium dodecyl sulfate polyacrylamide gel electrophoresis (SDS-PAGE) analysis detected the α2, α4, and α6 but not the α1, α3, α5, and α7* in the fractions co-eluted with α7^SR^ at approximately 21 min. The mass spectra of the α7^SR^/α2, α7^SR^/α4, and α7^SR^/α6 complexes under nondenaturing conditions showed a major ion series indicating their 1:7 hetero-octameric complexes with molecular masses of 225,164 ± 62, 229,435 ± 116, and 228,717 ± 124 Da (with theoretical masses of 225,230; 229,257; and 229,010 Da), respectively, as shown in Figure 6b. Regarding α2, MS peaks originating from the unbound α7 single ring were also observed, suggesting their weak interactions, as shown in Figure 6b. All these results indicate that α4 and α6, and to a lesser extent α2 but not the other α subunits, can interact with the disassembled α7 single ring in solution.

The cavitary surface of the homoheptameric ring of α7 exhibits unique charge distributions characterized by central negatively charged clusters surrounded by positively charged zones. The α2, α4, and α6 subunits commonly display positively and negatively charged clusters that are polarized near the vertex and at the base regions, respectively, of their triangle-shaped architecture; however, the negatively charged patch at the base region of α2 is smaller than those of α4 and α6, as shown in Figure S3. These charge complementarities may explain the selective accommodation of these α subunits in the cavity of the α7 homoheptameric ring. The double-ring cracking abilities of α4 and α6 may also be ascribed to their remarkably polarized charged clusters, although the structural basis of their transient interactions with the α7 double ring remains elusive.

It is plausible that the hetero-octameric complexes composed of the α7 and α4 (or α6) subunits cannot be re-associated into double rings because α4 as well as α6 occupies the central pore of the α7 homoheptameric ring, thereby sterically blocking inter-ring interactions. However, the hetero-octameric complexes are likely to be able to form complexes with unoccupied α7 homoheptameric rings, consequently encapsulating α4 and α6 within the α7 double-ring cage. One intriguing possibility is that these cages were detected as 14:1 α7/α4 and 14:1 α7/α6 complexes in the native mass spectra, as shown in Figure 5b.

### 2.4. Creation of Heterotetradecameric Double-Ring Structures of the Proteasomal Subunits

**Figure 7:**
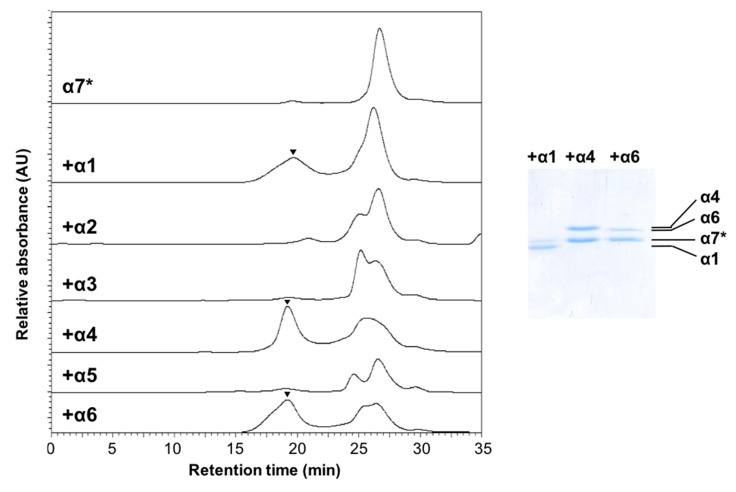
Exploration of possible formation of the α7 hetero-oligomeric complex mediated by proteasome α subunits. Size-exclusion chromatogram of α7* in the presence of α1–α6 subunits. The α1–α6 subunits (20 μM monomers) were mixed with an equimolar amount of α7* at 20 °C for 1 h, and the mixtures were subsequently analyzed by SEC. The peak position is indicated by an inverted triangle. SDS-PAGE of the purified α7*/α1, α7*/α4, and α7*/α6 complexes.

The self-assembling property of α7 suggests that proteasome formation involves some scrap-and-build mechanisms via which the α7 homotetradecamer is disassembled and the monomeric α7 subunit is integrated into the heteroheptameric α ring. To obtain insights into putative hetero-assembling processes involving α7, we used SEC to characterize possible interactions of the mutationally monomerized α7* with the other subunits, particularly α1 and α6, which flank α7 in the native heteroheptameric α-ring. Of note, the results indicated that α1, α4, and α6, but not the other α subunits, are co-assembled with α7*, giving rise to high–molecular-mass complexes of sizes comparable with that of the α7 homotetradecamer, as shown in Figure 7. The stoichiometry of the α7*/α1, α7*/α4, and α7*/α6 hetero-oligomeric complexes was estimated as 1:3.6, 1.2:1, and 2.5:1, respectively, based on the Coomassie Brilliant Blue (CBB)-staining SDS-PAGE results, as shown in Figure 7. 

**Figure 8:**
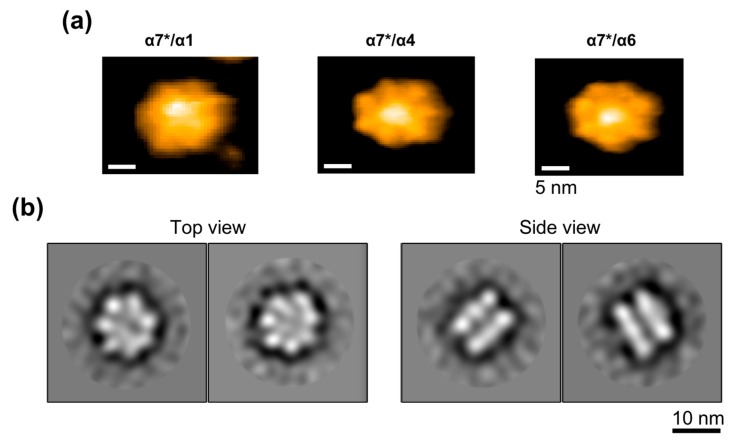
Structural characterization of the α7*/α4 and α7*/α6 hetero-oligomeric complexes. (**a**) AFM images of two typical orientations of the α7*/α1 (left), α7*/α4 (center), and α7*/α6 (right) complexes on bare mica. Scale bar: 5 nm. (**b**) Two-dimensional averaged image of an α7*/α4 particle subjected to single-particle negative-staining electron microscopy.

AFM data of the high-molecular-mass complex fraction of α7* with α4 or α6 identified tetradecameric particles of double-ring shape along with particles of single heptameric rings and oligomeric structures without ring shape, which presumably resulted from the disruption of the double-ring oligomers on the mica surface, as shown in Figure 8a. As for the α1/α7* complex, tetradecameric double rings and heptameric single rings were barely detected in the AFM observation possibly owing to their unstable structures on the mica surface. Instead, oligomeric structures were observed with a height of ~10 nm, which is comparable with those of the α7, α4/α7*, and α6/α7* tetradecamers (~9 nm). To obtain a higher-resolution structure, the α4/α7* hetero-oligomeric complex was subjected to negative-staining electron microscopy (EM), which also indicated that the complex had a double-ring tetradecameric structure, as shown in Figure 8b. 

It is puzzling that mixture of the artificially monomerized subunit (α7*) and the intrinsically monomeric subunit (α4 or α6) yields the heterotetradecameric double-ring architecture. One might assume that, for example, the α6–α7* interaction is stronger than the α6–α6 and α7*–α7* interactions. However, such more favorable residue pair(s) could not be found by simply comparing the amino acid residues at the intersubunit interfaces, i.e., at regions 2–5, based on the crystal structure of the α7 homotetradecamer, as shown in Figure S1 and Table S1. Rather, the crystallographic data highlight a small but significant difference between the α7–α7 contacting mode [25] and the α6–α7 interaction in the crystal structure of the human 20S proteasome, as shown in Figure S4 [28]. Consequently, the overall quaternary structure of the α7 homoheptameric ring is markedly different from that of the native heteroheptameric α ring. This implies that accumulation of the slightly different contact mode at the subunit interface results in geometric frustration of the formation of the circular quaternary structure, thereby causing deformation or disruption of the ring structure. Based on the data obtained in this study, we suggest that not only local structural complementarity at the subunit interfaces but also geometric consistency and/or structural adjustability in terms of the formation of the circular structure are factors that determine the heptamerization of the proteasome α subunits. The present study demonstrates that geometric frustration can be compromised by combining structurally homologous protomers with potential but imperfect ability to self-assemble, thereby providing insights for controlling the assembly and disassembly of the proteasomal subunits. Furthermore, our findings would provide clues for the design and creation of circular hetero-oligomers based on the homo-oligomeric structural frameworks.

## 3. Materials and Methods

### 3.1. Preparation of Wild-Type and Mutated Proteasome α Subunits

Human proteasome α6 short isoform [*PSMA1* (P25786); residues 1–263] and α7- [*PSMA3* (P25788); residues 1–255] subunits were expressed and purified as described previously [23,24,26]. Genes encoding proteasome α1 [*PSMA6* (P60900); residues 1–246] and α4 [*PSMA7* (P60900); residues 1–248] were subcloned into the *Nde*I and *Sal*I sites of pET28b (Merck Millipore, Burlington, MA, USA), whereas the α2 gene [*PSMA2* (P25787); residues 1–234] was subcloned into the *Nde*I and *Xho*I sites of pRSFDuet-1 vector (Merck Millipore). In addition, genes encoding α3 [*PSMA4* (P25789); residues 1–261] and α5 [*PSMA5* (P28066); residues 1–241] were subcloned into the *Bam*HI and *Xho*I or *Sal*I sites of modified pCold-I and pCold-GST vectors (TaKaRa Bio Inc., Kusatsu, Japan), respectively, which contain the TEV protease cleavage site preceding the target genes. Monomeric mutants of α7 (designated as α7* and α7^F14A^) were created via truncation of 22 N-terminal residues or introduction of the F14A mutation, respectively. The mutated α7 genes were subcloned into the *Nde*I and *Xho*I sites of the pET28b vector. In contrast, the single-ring mutant α7^SR^ was generated by introducing the S96D, S100D, F102R, and Y104R mutations using the wild-type construct in pRSFDuet-1. All expression plasmids were introduced into *Escherichia coli* BL21-CodonPlus (DE3)-RIL (Agilent Technologies, Santa Clara, CA, USA). 

For producing recombinant proteins, the *E. coli* cells harboring the expression plasmids were grown in Luria–Bertani medium containing 15 μg/mL kanamycin or 50 μg/mL ampicillin. The α7^SR^ mutant was purified as employed for the wild-type α7. Briefly, except for α2, the recombinant proteins were purified from the soluble fraction obtained by sonication and centrifugation. The resultant cell lysates were subjected to affinity chromatography [Ni^+^-charged Chelating Sepharose or Glutathione Sepharose 4B (GE Healthcare, Chicago, IL, USA)], and further purified using anion-exchange (RESOURCE Q, GE Healthcare) and size-exclusion (HiLoad 26/60 Superdex 75 or 200 pg; GE Healthcare) columns. The α2 was purified from the inclusion bodies and refolded according to standard dilution methods using a buffer containing 20 mM Tris-HCl (pH 8.0), 400 mM L-arginine, 250 mM NaSCN, 1 mM oxidized glutathione, and 5 mM reduced glutathione. The refolded protein was further purified using a HiLoad 26/60 Superdex 75pg column (GE Healthcare). 

### 3.2. Determination of Molecular Mass

The molecular masses of the human wild-type proteasome α subunits (α1–α7) and the mutated α7 proteins (α7*, α7^F14A^, and α7^SR^) were estimated using SEC and native MS. In SEC, the samples (0.3–10 μM) were loaded onto a Superose 6 increase 10/300 GL column (GE Healthcare) with 20 mM Tris-HCl (pH 8.0) and 150 mM NaCl at a flow rate of 0.75 mL/min. For calibrating the column, ribonuclease A (13.7 kDa), chymotrypsinogen A (25 kDa), ovalbumin (43 kDa), bovine serum albumin (67 kDa), conalbumin (75 kDa), aldolase (158 kDa), ferritin (440 kDa) (GE Healthcare), and blue dextran 2000 were used. The elution profiles were recorded as absorbance values at 280 nm. 

In native MS, buffer exchange of the purified α1–α7 proteins (30–50 μM monomers) was performed using 150 mM ammonium acetate (pH 6.8–8.0) with a Bio-Spin 6 column (Bio-Rad, Hercules, CA, USA). The buffer-exchanged samples (5–20 μM monomers) were immediately subjected to nanoflow electrospray ionization MS analysis with gold-coated glass capillaries made in house. Approximately 2–5 µL samples were loaded for each measurement. Buffer-exchanged α4 and α6 (4 μM monomers) were mixed with α7 (2 μM tetradecamer) at 20 °C for 1 h and subsequently analyzed using native MS. In contrast, α4 and α6 (4 μM monomers) were mixed with α7^SR^ mutant (4 μM heptamer) and incubated as employed for wild-type α7. As for α2, the sample was incubated with α7^SR^ beforehand, and the buffer exchange was carried out for the mixture, and then subjected to native MS measurements. Spectra were acquired on a SYNAPT G2-S*i* HDMS mass spectrometer (Waters, Manchester, UK) in the positive ionization mode, as previously described [25]. Spectrum calibration was performed using 1 mg/mL of cesium iodide and analysis was performed using the Mass Lynx software (Waters). 

### 3.3. AFM

For AFM sample preparation, the α1, α4, or α6 (20 μM monomers) was mixed with an equal molar amount of α7* at 20 °C for 1 h, and subsequently fractionated by SEC. The high-molecular-mass complex fractions were subjected to the AFM analysis. AFM was performed using a laboratory-constructed apparatus with cantilevers (7 μm long, 2 μm wide, and 90 nm thick) at room temperature [29]. Typical values of the spring constant, resonant frequency, and quality factor of the cantilever in an aqueous solution are approximately 0.2 N/m, 800 kHz, and 2, respectively. In AFM imaging, the free and set-point oscillation amplitudes were set to approximately 1.5 nm and 90% of the former, respectively. All samples were applied to either bare mica in 20 mM Tris-HCl (pH 8.0) with 150 mM NaCl, as previously described [26]. 

### 3.4. EM

The protein samples for EM measurement were prepared using the same protocol as in the AFM analysis. Negative-staining EM was performed using a conventional protocol, as previously described [30]. EM imaging of the α4/α7* hetero-oligomeric complex was performed at room temperature using a JEOL JEM 2200FS electron microscope (JEOL Ltd., Tokyo, Japan) equipped with a field emission gun operating at an acceleration voltage of 200 kV. A total of 40 images were obtained using a DE20 direct detection camera (Direct Electron LP, San Diego, CA, USA) at a detector magnification of 50,000 with an energy slit width of 20 eV using the low-dose mode. The image size was set to 1.09 Å per pixel on the camera. After subjection of motion collection with the DE_process_frames.py script, the obtained images were processed with Relion 2.0 software [31]. Subsequently, 1,534-particle images were extracted from the 40 images and subjected to two-dimensional classification after sorting with cross-correlation coefficients.

## Supplementary Materials

Supplementary Materials can be found at https://www.mdpi.com/1422-0067/20/9/2308/s1.

## Abbreviations

AFM	atomic force microscopy
CBB	Coomassie Brilliant Blue
EM	electron microscopy
MS	mass spectrometry
*m*/*z*	mass-to-charge ratio
PDB	Protein Data Bank
SDS-PAGE	sodium dodecyl sulfate polyacrylamide gel electrophoresis
SEC	size-exclusion chromatography
SR	single-ring
