# Supplementary material for: Mutational and Combinatorial Control of Self-Assembling and Disassembling of Human Proteasome α Subunits
Source: Int J Mol Sci. 2019 May 9;20(9):2308. doi: 10.3390/ijms20092308 (PMC6539845; doi:10.3390/ijms20092308)
Supplement: Supplementary file 1 [file ijms-20-02308-s001.pdf]

Supplementary Material

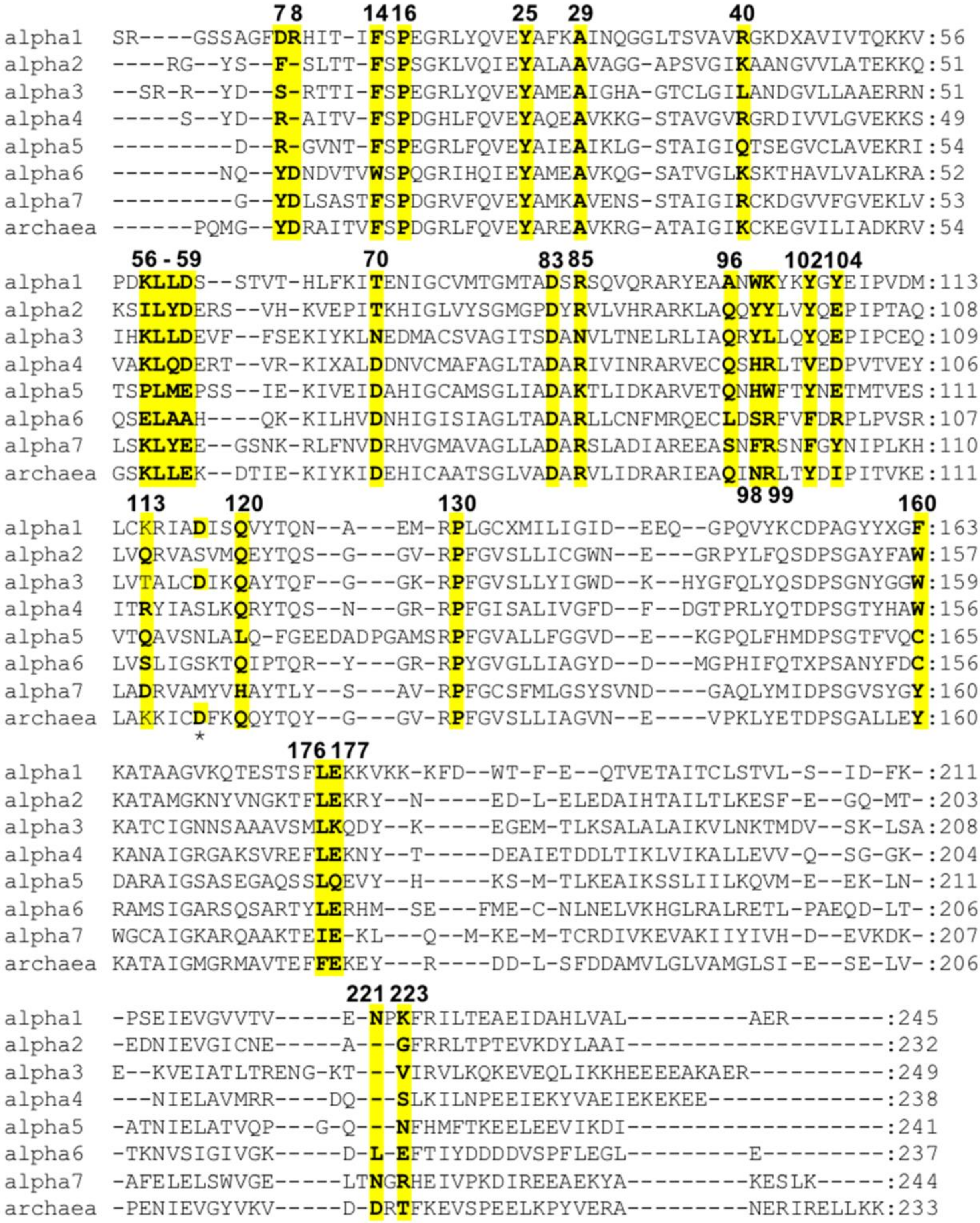

Figure S1. Structure-based sequence alignment of the human proteasome α1–α7 subunits together with the archaeal α subunit (*Archaeoglobus fulgidus*)

The amino acid sequences are aligned using the program MATRAS [26]. Residues involved in intersubunit *cis* and *trans* interactions of the human α7 homotetradecamer (PDB code: 5DSV) [16] and 20S proteasome (5LE5) [22] are highlighted in yellow.

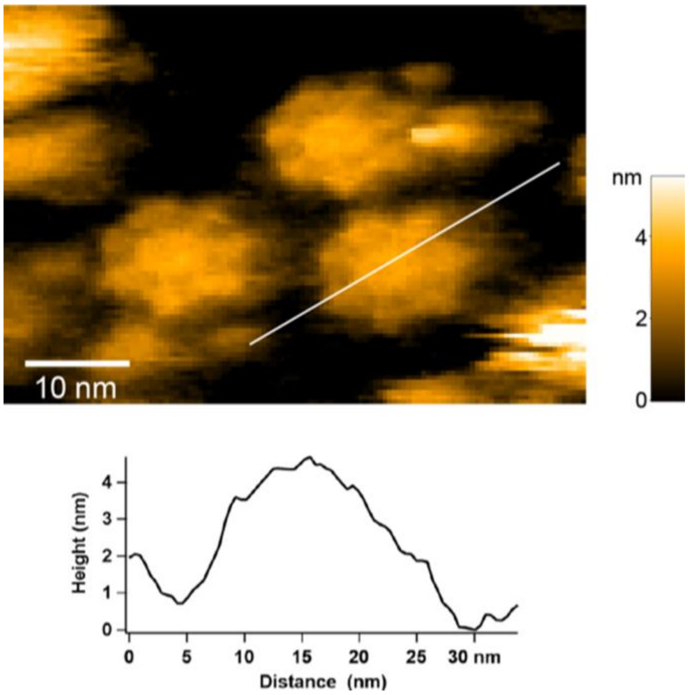

Figure S2. Structural characterization of α7^{SR}

An AFM image of typical orientations of the α7^{SR} and its cross-sectional profile observed on bare mica.

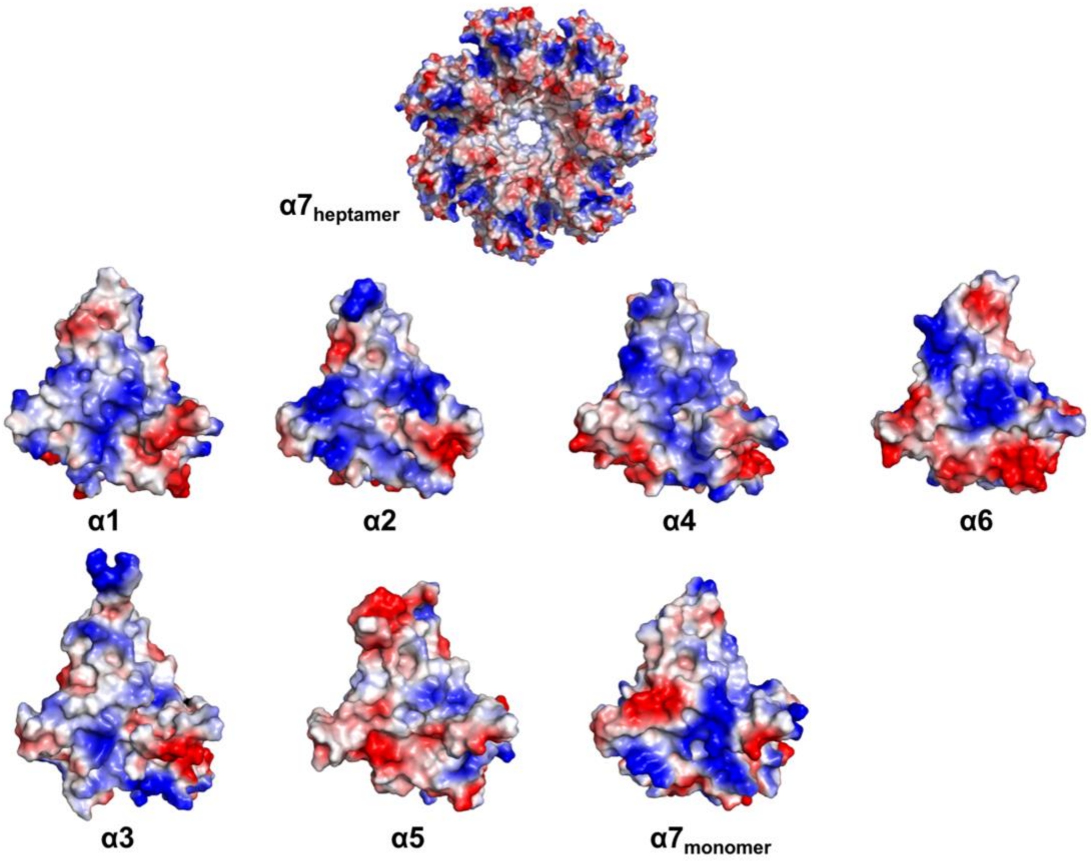

Figure S3. Surface potential representation of the human proteasome α subunits

An α7 heptameric ring extracted from the α7 tetradecamer along with the α1–α6-subunits derived from the 20S proteasome is shown. The surface models are colored according to the electrostatic surface potential (blue, positive; red, negative).

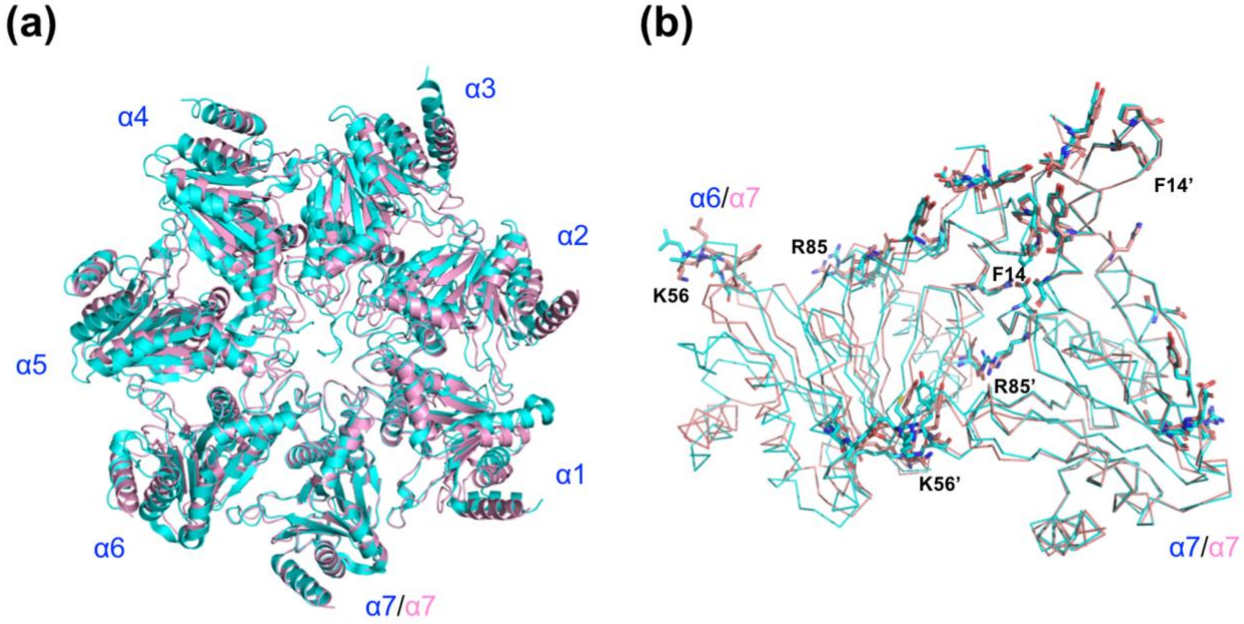

Figure S4. Structural comparison of the proteasomal α-subunit interactions

(a) Superimposition of the overall structures of homoheptameric (pink) and heteroheptameric (cyan) α-rings derived from the human α7 homotetradecamer and the 20S proteasome, respectively, are represented. (b) Intersubunit interactions between the α7–α7 and α6–α7 interfaces are shown. Residues involved in α7–α7 and α6–α7 interactions are shown in the stick model. In the α7–α7 interacting residues, Phe14, Lys56, and Arg85 in regions 1–3 are labeled as the representatives.

Table S1. Summary of intersubunit contacting residue pairs in the crystal structures of the human α7 homotetradecamer and the archaeal α homoheptamer together with the corresponding residues of the human α1–α6 subunits.

	cis							*trans*			
	Region 1 (Mol1/Mol2)		Region 2 (Mol1/Mol2)			Region 3 (Mol1/Mol2)		Region 4 (Mol1/Mol3)		Region 5 (Mol1/Mo4)	
α1	R11/D10	Y27/P18	A28/F16	K59/E180	L60/L179	D58/R43	D86/Q123	T84/D120	T73/K102	K226/N224	Y107/Y107
α2	-/F8	Y24/P15	A28/F13	I55/E175	L56/L174	D58/K39	D82/Q119	R84/Q112	T69/Y98	G217/-	E103/E103
α3	-/S7	Y23/P14	A27/F12	K54/K176	L55/L175	D57/L38	D82/Q119	N84/D116	N69/L98	V224/-	E103/E103
α4	-/R5	Y21/P12	A25/F10	K52/E173	L53/L172	D55/R36	D79/Q116	R81/R109	D66/R95	S216/-	D100/D100
α5	-/R10	Y26/P17	A30/F15	P57/Q182	L58/L181	E60/Q41	D84/L121	K86/Q114	D71/W100	N225/-	E105/E105
α6	D7/Y6	Y24/P15	A28/Y6	E55/E173	L56/L172	A58/K39	D80/Q117	R82/S110	D67/R96	E220/L219	R101/R101
α7	D8/Y7	Y25/P16	A29/ F14	K56/E177	L57/I176	E59/R40	D83/H120	R85/D113	D70/R99	R223/N221	Y104/Y104
α	D9/Y8	Y26/P17	A30/F15	K57/E176	L58/F175	E60/K41	D84/Q121	R86/D118	D71/R100	T220/D218	I105/I105
